# Mobile App–Assisted Self-Monitoring of Blood Glucose in Type 2 Diabetes in Ningbo, China: 12-Month Retrospective Cohort Study

**DOI:** 10.2196/65919

**Published:** 2025-09-02

**Authors:** Xujia Ma, Kaushik Chattopadhyay, Miao Xu, Li Li, Jialin Li

**Affiliations:** 1 Department of Endocrinology and Metabolism, The First Affiliated Hospital of Ningbo University, Building 2, 22nd Fl., 59 Liuting Street, Ningbo, 315010, China, 86 13780008400; 2 Lifespan and Population Health, School of Medicine, University of Nottingham, Nottingham, United Kingdom

**Keywords:** blood glucose, China, cohort study, mobile app, self-management, self-monitoring, type 2 diabetes

## Abstract

**Background:**

Self-monitoring of blood glucose (SMBG) is recommended in clinical practice guidelines, including those in China, as part of patient education, self-management, and empowerment. With technological advancements, telecommunication technologies are now used for telemonitoring in health care. Mobile apps have become a practical tool for SMBG among patients with type 2 diabetes mellitus (T2DM). However, the long-term effectiveness of this approach in real-world practice requires further exploration.

**Objective:**

The study aims to determine the effectiveness of mobile app–assisted SMBG in improving glycemic control in patients with T2DM at 12 months, in addition to standard care, in Ningbo, China.

**Methods:**

In this retrospective cohort study, adults with T2DM who registered at the National Metabolic Management Center, Ningbo, for the first time between September 1, 2019, and June 30, 2022, and received standardized diabetes management were included. The study compared 2 groups: those who opted for mobile app–assisted SMBG and those who did not. Propensity score matching matched the mobile app–assisted SMBG group with the control group based on similar baseline characteristics. Glycemic control–related outcomes were compared at 12-month follow-up. Linear and logistic regression models were used to estimate mean differences and odds ratios (ORs) along with 95% CIs, respectively, and adjustments were made for baseline characteristics.

**Results:**

A total of 160 patients (80 in each group) were included in the study. In the mobile app–assisted SMBG group, the median (IQR) frequency of blood glucose monitoring was 0 (0-2) times per week, with 28% (22/80) monitoring their blood glucose at least twice per week, and the app usage frequency was 1 (0-3) time per week, with 40% (32/80) logging in at least twice per week. There were no statistically significant differences observed between the mobile app–assisted SMBG group and the control group in glycemic control outcomes at 12 months. Specifically, the results showed no significant difference in (1) fasting blood glucose and glycosylated hemoglobin levels (mean difference −0.17 mmol/L, 95% CI −0.85 to 0.51 mmol/L; *P*=.62 and −0.12%, 95% CI −0.58% to 0.33%; *P*=.59, respectively) and (2) the proportion of patients achieving or maintaining fasting blood glucose at <7 mmol/L and glycosylated hemoglobin at <7% (OR 0.89, 95% CI 0.46-1.73; *P*=.74 and OR 0.91, 95% CI 0.44-1.88; *P*=.79, respectively).

**Conclusions:**

In a real-world cohort of patients with T2DM in Ningbo, China, mobile app–assisted SMBG did not lead to statistically significant improvements in glycemic control at 12 months. This suggests that in a well-resourced setting, standard care alone may be relatively effective. However, opportunities for further improvement remain. The lack of observed benefit may be due to process-related issues, such as suboptimal engagement with the intervention. Addressing these challenges should be a focus of future research.

## Introduction

### Background

China has the highest prevalence of type 2 diabetes mellitus (T2DM) in the world, with approximately 130 million (11%) adults affected by T2DM [1]. T2DM is a complex metabolic disorder with significant implications for health, society, and the economy [2,3]. The persistent elevation of blood glucose levels in T2DM is associated with numerous complications, diminished quality of life, and heightened mortality rates [4-6]. T2DM clinical practice guidelines recommend adequate glycemic control, aiming for a fasting blood glucose level of <7 mmol/L and glycosylated hemoglobin (HbA_1c_) level of <7% in most patients [7,8].

The role of self-monitoring of blood glucose (SMBG) in patients with T2DM, especially those not on insulin or at lower risk of hypoglycemia, remains debatable [9-13]. However, many clinical practice guidelines, including those in China, recommend SMBG as part of patient education, self-management, and empowerment [14,15]. The most common method for SMBG is the finger-stick capillary blood glucose test using a glucometer [14]. The frequency of SMBG depends on several factors, including the patient’s blood glucose level, treatment regimen, and risk of hypoglycemia [14].

With technological advancements, various telecommunication technologies are now used for telemonitoring in health care [16]. Systematic reviews suggest that telemonitoring interventions may effectively improve glycemic control in patients with T2DM, although more primary studies are needed to evaluate the effectiveness of specific intervention delivery modes [17,18]. In China, mobile apps are used for SMBG in real clinical practice, including for patients with T2DM [19]. However, the effectiveness of this approach, especially in real-world practice and over the long term, required further exploration [20].

Although Ningbo is one of China’s most economically developed cities [21] and had a T2DM prevalence of approximately 10% in 2018 [22], glycemic control among patients has remained suboptimal. For example, a community-based study of patients with T2DM in Ningbo reported an average HbA_1c_ level of 59.6 mmol/mol (7.6%) [23]. Similarly, a previous study conducted at our tertiary care diabetes center—before the implementation of standardized care under the National Metabolic Management Center (MMC) program—found that only half of the patients achieved the HbA_1c_ target of <7% after 6 months of treatment [24]. These findings reinforce that despite the availability of health care resources, glycemic targets are frequently unmet in routine clinical practice.

### Objectives

This study aimed to evaluate whether the addition of mobile app–assisted SMBG to standard care could improve glycemic control over 12 months in patients with T2DM in Ningbo, China.

## Methods

### Study Design, Location, Population, and Baseline Characteristics

This retrospective cohort study used data from the MMC at The First Affiliated Hospital of Ningbo University. This MMC is the largest and main center in Ningbo and is one of the two provincial-level MMCs in the Zhejiang Province [25]. Adults (18-75 y) with T2DM who registered at this MMC for the first time between September 1, 2019, and June 30, 2022, and received standardized diabetes management were included. Baseline characteristics included sociodemographic information (age, sex, education, and household income) and clinical information (such as duration of T2DM [self-reported], blood glucose levels, and insulin regimen).

### Exposure

All patients received standard care under the MMC program, which involved standardized diabetes management. SMBG and its importance were explained during the first visit, as recommended by the Chinese T2DM clinical practice guidelines [7]. Some patients voluntarily purchased a mobile app–assisted glucometer for self-monitoring (EZ-8 SIM Blood Glucose Meter with the Huayi Glucose Manager app, SINOMEDISITE, China), forming the mobile app–assisted SMBG group in the study. The device and app bundle cost approximately 200 RMB (about US $28), including the initial supply of test strips. The hospital had no financial or contractual ties to the manufacturer and did not offer direct technical support. Clinicians introduced the app during routine visits as a voluntary, supplementary tool. Those who did not opt for the app-assisted SMBG formed the control group.

**Figure 1.**
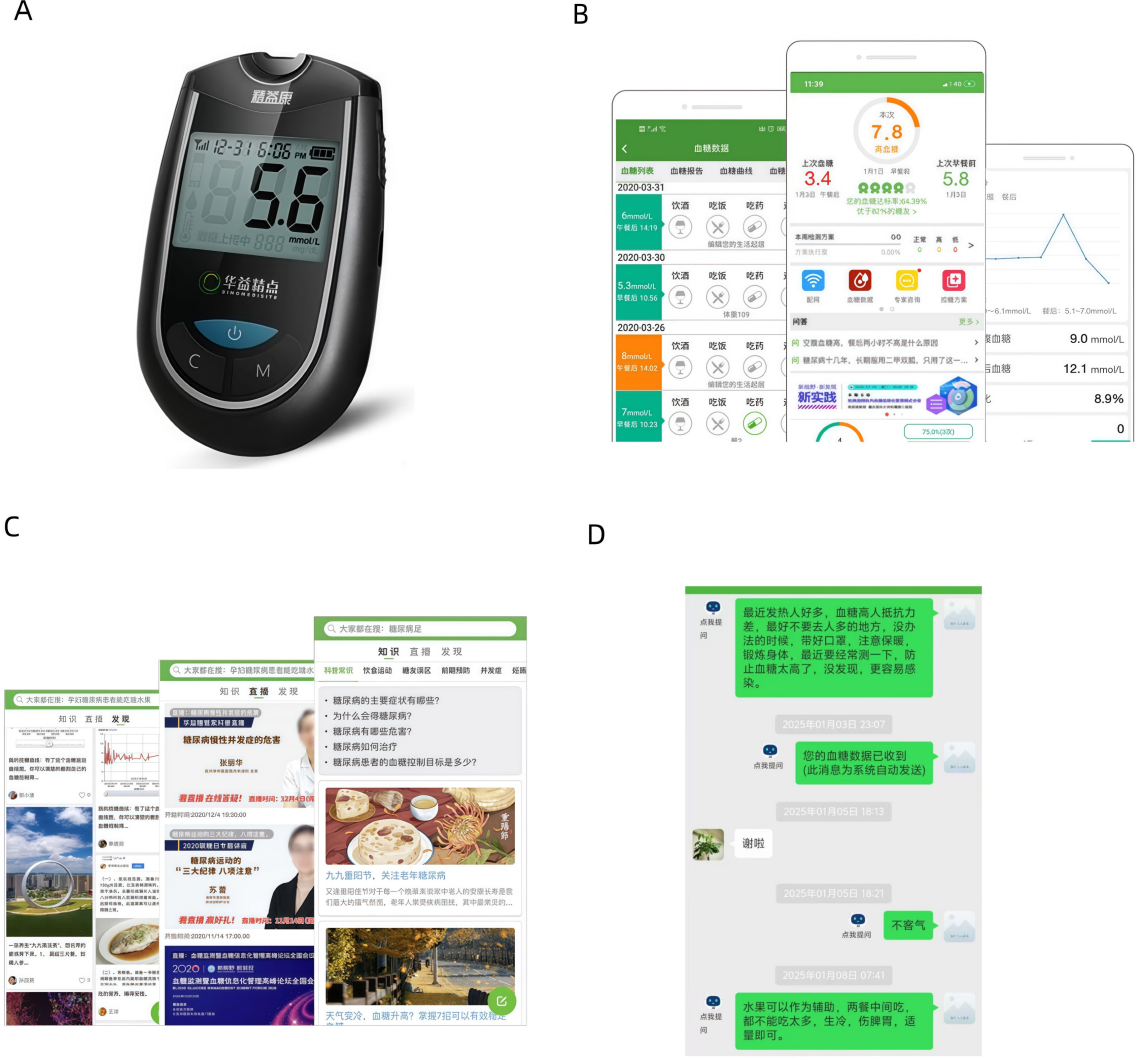
(A) EZ-8 SIM blood glucose meter. (B) Huayi Glucose Manager app blood glucose data visualization interface. (C) App educational content interface. (D) App clinician communication interface.

The EZ-8 SIM Blood Glucose Meter is a compact device designed to measure capillary blood glucose levels, displaying results in millimoles per liter on a digital screen (see Figure 1A). It includes basic navigation buttons and connects to the Huayi Glucose Manager app via Bluetooth for data transmission.

The Huayi Glucose Manager app is a mobile-based telemonitoring platform intended to support SMBG in patients with T2DM. Blood glucose readings are automatically transmitted from the glucometer to the app and categorized as in-range (green), high (yellow), or low (red), with accompanying graphical displays (see Figure 1B). These features are designed to assist patients and clinicians in tracking glycemic trends in real time.

Patients can interact with the app by logging in multiple times per week. According to Chinese guidelines, SMBG is recommended twice weekly for patients on oral antidiabetic drugs (OADs), whereas those receiving insulin therapy are advised to monitor blood glucose levels more frequently throughout the day [7]. The app includes additional features intended to support self-management, such as prerecorded educational sessions (see Figure 1C) covering T2DM, complications associated with T2DM, and diet and lifestyle programs. Users can set lifestyle goals, track progress, and view changes in blood glucose levels corresponding to behavioral modifications. The app also allows direct communication between patients and clinicians (see Figure 1D).

Clinicians can access patient data and blood glucose readings via a QR code linked to the app, facilitating tailored advice, monitoring reminders, and secure communication. The app further provides a live-stream feature, enabling clinicians to conduct patient education sessions related to T2DM.

### Outcomes

Outcomes at 12-month follow-up included fasting blood glucose levels, the proportion of patients achieving or maintaining a fasting blood glucose level of <7 mmol/L, HbA_1c_ levels, and the proportion of patients achieving or maintaining an HbA_1c_ level of <7%. Fasting blood glucose was assessed using the glucose oxidase method. HbA_1c_ was estimated using the high-performance liquid chromatography method with the D-10 Haemoglobin Analyzer (Bio-Rad).

### Ethical Considerations

This study was approved by the Research Ethics Committee of The First Affiliated Hospital of Ningbo University (2019-R057). All patients, irrespective of future group assignment, provided written informed consent at the time of registration at the MMC, permitting the use of their routinely collected clinical data for research purposes. No additional consent was sought after the matching process, as this study involved retrospective analysis of anonymized data. To ensure patient privacy and confidentiality, all personal identifying information (such as names, hospital record numbers, and contact information) was removed from the dataset before analysis. The research team only had access to the fully anonymized data. Participants did not receive any compensation for their data being included in this study.

### Statistics

Among patients with baseline and 12-month follow-up data on blood glucose levels (80 in the mobile app–assisted SMBG group; 1490 in the control group), propensity score matching was performed using R (version 4.1.2; *MatchIt* package, R Foundation for Statistical Computing). This process paired each patient in the mobile app–assisted SMBG group with a patient in the control group based on similar baseline characteristics [26]. Once a match was made, the patients were not reconsidered for further matching. Absolute standardized differences were calculated for baseline characteristics, with a value of <10% generally regarded as a marker of acceptable balance [27]. A multivariable regression model was used to calculate the propensity score. A 1:1 ratio matching algorithm with nearest-neighbor matching and no replacement was used, with a caliper width of 0.1 SD.

For categorical data, numbers and percentages were calculated. For continuous data, summary measures (mean or median and spread) were calculated depending on data distribution. Linear and logistic regression models were used to estimate unadjusted and adjusted mean differences (MDs) and odds ratios (ORs) along with 95% CIs, respectively. Adjustments were made for baseline characteristics. Data were analyzed using SPSS Statistics for Windows (version 26.0; IBM Corp).

## Results

A total of 160 patients (80 in each group) were included in the matched cohort. At baseline, the mean (SD) age of patients was 47.13 (10.56) years, with 66 (41.3%) patients being female. The mean (SD) values for BMI, fasting blood glucose, and HbA_1c_ levels were 25.88 (4.06) kg/m², 9.17 (2.96) mmol/L, and 8.54% (2.36%), respectively. Regarding diabetes treatment, 71.9% (115/160) of the patients were treated with OADs alone, whereas 28.1% (45/160) of the patients were on insulin regimens, either alone or in combination with OADs. Diabetic microvascular complications were present in 53.8% (86/160) of the patients, whereas diabetic macrovascular complications were present in 4.4% (7/160) of the patients. The baseline characteristics of the 2 groups are shown in Table 1 (for characteristics before matching, see Table S1 in Multimedia Appendix 1). The 2 groups were similar in most baseline characteristics, with absolute standardized differences <10%, except for the duration of T2DM, which had a difference of 15.57%.

**Table 1.** Baseline characteristics of patients in the matched cohort (N=160).

	Mobile app–assisted SMBG^a^ group (n=80)	Control group (n=80)	Absolute standardized difference (%)	*P* value
Sociodemographic characteristics				
Age (years), mean (SD)	46.79 (10.04)	47.48 (11.10)	6.85	.68
Sex, n (%)				>.99
Male	47 (58.75)	47 (58.75)	0	
Female	33 (41.25)	33 (41.25)	0	
Education, n (%)				>.99
<High school	34 (42.50)	34 (42.50)	0	
≥High school	46 (57.50)	46 (57.50)	0	
Household income (RMB^b^), n (%)				.46
≤100,000	29 (36.25)	31 (38.75)	5.20	
>100,000‐300,000	30 (37.50)	29 (36.25)	2.58	
>300,000	18 (22.50)	20 (25.00)	5.99	
Unknown	3 (3.75)	0 (0)	19.74	
Clinical characteristics				
Duration of T2DM^c^ (months), median (IQR)	38.50 (15.50-69.75)	52.50 (12.00-107.00)	15.57	.10
Fasting blood glucose (mmol/L), mean (SD)	9.20 (2.94)	9.14 (3.00)	2.06	.90
HbA_1c_^d^ (%), mean (SD)	8.57 (2.33)	8.50 (2.41)	3.01	.85
Insulin regimen, n (%)				.60
No	59 (73.75)	56 (70.00)	8.52	
Yes	21 (26.25)	24 (30.00)	8.52	

a SMBG: self-monitoring of blood glucose.

b RMB: Chinese Yuan (1 RMB=US $0.14).

c T2DM: type 2 diabetes mellitus.

d HbA_1c_: glycosylated hemoglobin.

In the mobile app–assisted SMBG group, the median (IQR) frequency of blood glucose monitoring was 0 (0-2) times per week, with 28% (22/80) of patients monitoring their blood glucose at least twice per week. The median (IQR) app usage frequency was 1 (0-3) time per week, with 40% (32/80) of patients logging in at least twice per week.

**Figure 2.**
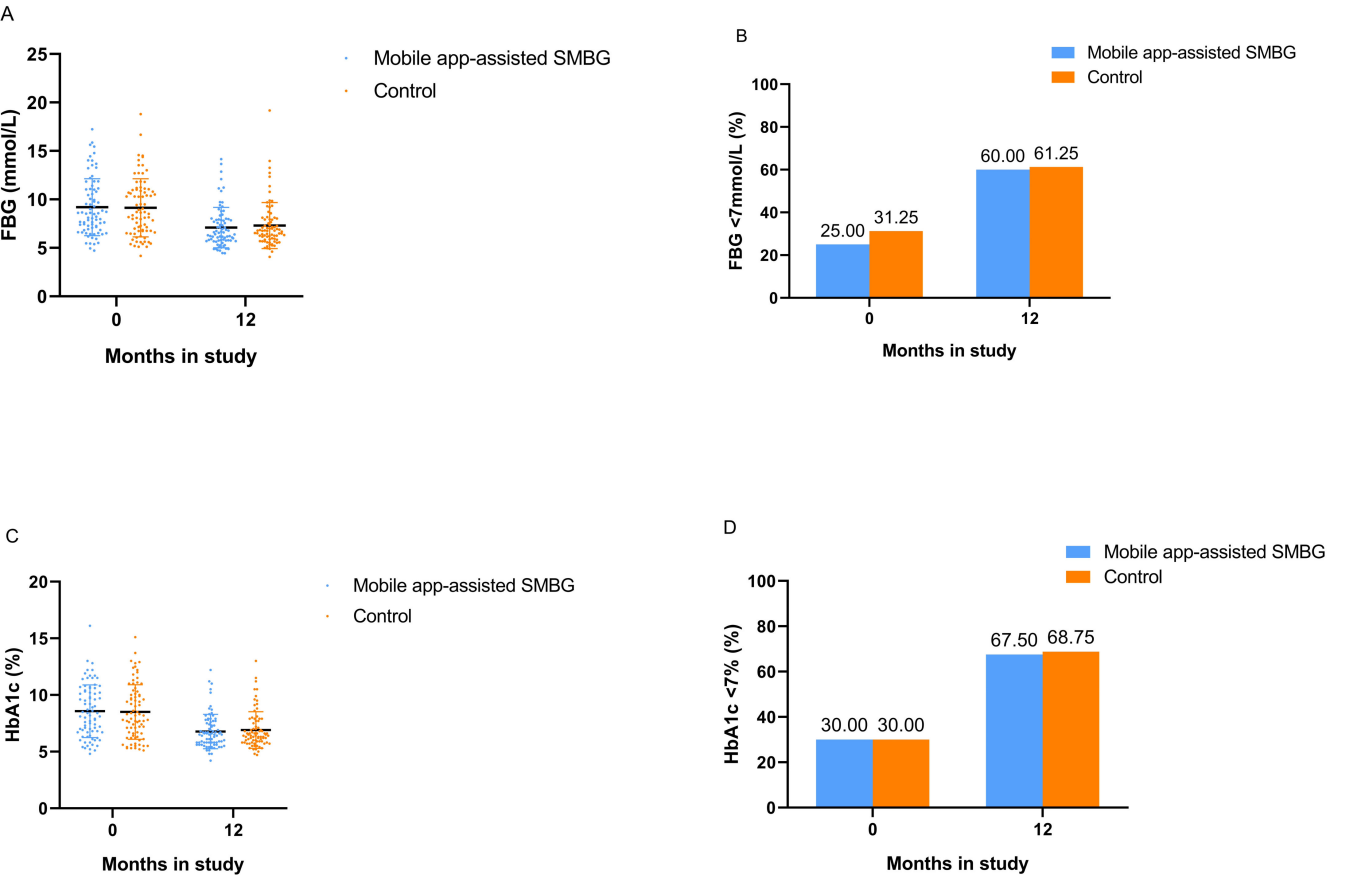
Unadjusted outcomes at 12 months. (A) FBG levels. (B) Proportion of patients achieving or maintaining an FBG level of <7 mmol/L. (C) HbA_1c_ levels. (D) Proportion of patients achieving or maintaining an HbA_1c_ level of <7%. FBG: fasting blood glucose; HbA_1c_: glycosylated hemoglobin; SMBG: self-monitoring of blood glucose.

Figure 2 shows unadjusted outcomes at 12 months. The mean (SD) fasting blood glucose in the mobile app–assisted SMBG and control groups was 7.09 (2.09) mmol/L and 7.31 (2.38) mmol/L, respectively (MD −0.21 mmol/L, 95% CI −0.91 to 0.49 mmol/L; *P*=.55). The proportion of patients achieving or maintaining a fasting blood glucose level of <7 mmol/L in the mobile app–assisted SMBG and control groups was 60% (48/80) and 61% (49/80), respectively (OR 0.95, 95% CI 0.50-1.79; *P*=.87). The mean (SD) of HbA_1c_ in the mobile app–assisted SMBG and control groups was 6.78% (1.51%) and 6.91% (1.61%), respectively (MD −0.13%, 95% CI −0.62% to 0.36%; *P*=.59). The proportion of patients achieving or maintaining an HbA_1c_ level of <7% in the mobile app–assisted SMBG and control groups was 68% (54/80) and 69% (55/80), respectively (OR 0.94, 95% CI 0.49-1.84; *P*=.87).

Table 2 reports adjusted outcomes at 12 months. There were no statistically significant differences observed between the mobile app–assisted SMBG group and the control group in glycemic control outcomes at 12 months. Specifically, the results showed (1) no significant difference in fasting blood glucose and HbA_1c_ levels (MD −0.17 mmol/L, 95% CI −0.85 to 0.51 mmol/L; *P*=.62 and −0.12%, 95% CI −0.58% to 0.33%; *P*=.59, respectively) and (2) no significant difference in the proportion of patients achieving or maintaining a fasting blood glucose level of <7 mmol/L and an HbA_1c_ level of <7% (OR 0.89, 95% CI 0.46-1.73; *P*=.74 and OR 0.91, 95% CI 0.44-1.88; *P*=.79, respectively). An additional data analysis at 6 months is provided in Table S2 in Multimedia Appendix 1. At 6 months, the mobile app–assisted SMBG group showed a reduction in HbA_1c_ compared with the control group (adjusted MD −0.43%, 95% CI −0.86% to 0%; *P*=.05), reaching borderline statistical significance. No significant differences were observed for fasting blood glucose levels or target achievement rates.

**Table 2.** Adjusted outcomes at 12 months^a^.

	Adjusted MD^b^/OR^c^ (95% CI)		*P* value
Fasting blood glucose (mmol/L)	−0.17 (−0.85 to 0.51)		.62
Fasting blood glucose <7 mmol/L			
Control	Ref		—^d^
Mobile app–assisted SMBG^e^	0.89 (0.46-1.73)		.74
HbA_1c_^f^ (%)	−0.12 (−0.58 to 0.33)		.59
HbA_1c_ <7%			
Control	Ref		—
Mobile app–assisted SMBG	0.91 (0.44-1.88)		.79

a Adjusted for age, sex, education, household income, duration of type 2 diabetes mellitus, baseline fasting blood glucose/HbA_1c_, and insulin regimen.

b MD: mean difference.

c OR: odds ratio.

d Not applicable.

e SMBG: self-monitoring of blood glucose.

f HbA_1c_: glycosylated hemoglobin.

## Discussion

Our study assessed the long-term effectiveness of mobile app–assisted SMBG in improving glycemic control in patients with T2DM at 12 months, alongside standard care, in Ningbo, China. Although the mobile app–assisted SMBG group showed lower fasting blood glucose and HbA_1c_ levels compared with the control group, these differences were not statistically significant. Similarly, the proportion of patients achieving or maintaining a fasting blood glucose level of <7 mmol/L and an HbA_1c_ level of <7% was lower in the intervention group; however, these differences did not reach statistical significance.

Despite the lack of statistical significance, our findings offer valuable insights into the real-world application of mobile app–assisted SMBG. While standard care in this well-resourced setting has been shown to be effective [28-30], opportunities for further improvement remain. In our study, the supplementary mobile app–assisted SMBG did not yield clear benefits, likely due to process-related factors such as limited engagement, rather than a ceiling effect. Addressing these implementation challenges should be a key focus of future research. Specifically, previous studies have demonstrated that standard care at MMC effectively manages glycemic control in patients with T2DM [28-30]. Similarly, in our study, at baseline (ie, the point of registration at MMC for standardized diabetes care), the mean fasting blood glucose in both groups exceeded 9 mmol/L. By 12 months, this had decreased to approximately 7 mmol/L. Likewise, the mean HbA_1c_ at baseline was ≥8.5%, which declined to <7% at 12 months. These findings suggest that MMC’s standardized care may be effective in improving glycemic control in individuals with T2DM. Patients in the intervention group monitored their blood glucose less frequently than the approximately 2 times per day typically associated with meaningful glycemic improvements [31], which may have limited the intervention’s impact. The supplementary 6-month analysis showed a borderline significant reduction in HbA_1c_ in the intervention group, but this was not sustained at 12 months. This trend likely reflects a decline in engagement over time—a common challenge in digital health interventions—and aligns with previous studies reporting reduced effectiveness of mobile app–assisted SMBG as usage wanes [32-35]. Furthermore, given that our study participants had a median diabetes duration of 3 to 4 years, they were likely not newly diagnosed and may have already been familiar with SMBG through standard care, potentially reducing the added benefit of mobile app–assisted SMBG. Research indicates that mobile app–assisted SMBG is most beneficial for patients newly diagnosed with T2DM, particularly within the first 3 to 6 months, when treatment adjustments are most critical [34,36,37].

Systematic reviews examining factors associated with the uptake, engagement, and retention of mobile apps, including those used for self-management of chronic conditions such as diabetes, have identified a range of influences aligned with the Capability, Opportunity, Motivation for Behavior model. These include challenges such as usability challenges, technical difficulties, lack of customizable features, absence of supportive functionalities (eg, reminders or feedback), and limited perceived usefulness of the apps [38-40]. In addition, a previous study evaluating Chinese mobile apps for diabetes self-management identified concerns related to app quality, functionality, and features and recommended improvements through co-design involving researchers, health care professionals, and target users [41]. While it is possible that the developers of the mobile app used in our study received some informal feedback from key stakeholders, we are not aware of any systematic co-development approach undertaken.

To the best of our knowledge, this is the first study to assess the long-term (12 mo) effectiveness of mobile app–assisted SMBG in a real-world cohort of patients with T2DM in Ningbo, China. Unlike randomized controlled trials, which are rigorously controlled but may not fully reflect the complexities of real-world clinical settings [42,43], our study leveraged real-world data from routine clinical practice within a high-quality treatment environment. Propensity score matching was used to create a balanced control group and address potential confounders, with known confounders further adjusted for in the analysis. Despite this, the absolute standardized difference in diabetes duration between groups remained above the accepted 10% threshold, with the intervention group having a shorter average duration. This imbalance may have conferred an advantage, as shorter diabetes duration is typically associated with better glycemic control [24], and mobile app interventions tend to be more effective in individuals with more recent diagnoses [44]. Nevertheless, we did not observe statistically significant improvements in glycemic control in the intervention group, even with this potential bias in its favor.

Several other limitations should also be acknowledged. First, the small sample size reduced statistical power, which may have limited our ability to detect subtle differences. As this was a retrospective analysis of existing data, no formal sample size calculation was performed. Instead, we included all eligible patients (n=1570) and applied propensity score matching to design this retrospective cohort study (n=160). Because patients voluntarily purchased the mobile app–assisted glucometers, the intervention group may have differed from the control group, introducing potential confounding factors. Apart from the app development and engagement issues discussed earlier, the study encountered additional process-related limitations due to restricted dataset access granted by the app developers for research purposes. First, although we reported the overall frequency of app engagement by patients, including SMBG, the dataset available to us did not include longitudinal data needed to evaluate usage patterns over time. This constrained our ability to assess whether declining engagement may have contributed to the waning effectiveness of the intervention. In addition, we did not have access to data on how clinicians and patients interacted with specific app features—such as doctor-patient communication, delivery of clinical advice, or patient self-management—limiting insights into the mechanisms of engagement. Safety data were also unavailable in this retrospective study, as they were not routinely collected, limiting the comprehensive evaluation of the intervention. Because the study was conducted in a highly specialized, resource-intensive setting, the generalizability of our findings to lower-resource environments may be limited. There are inherent trade-offs in real-world observational studies, which prioritize practical applicability and relevance over the controlled conditions typically found in randomized controlled trials. Future large-scale, prospective studies are needed to evaluate high-quality mobile app–assisted SMBG interventions, incorporating strategies to optimize engagement and focusing on both long-term effectiveness and safety. Causal inference frameworks such as the Bradford Hill criteria or the 9 principles for determining causality in epidemiology should be applied to strengthen the evidence base [45].

In conclusion, in a real-world cohort of patients with T2DM in Ningbo, China, mobile app–assisted SMBG did not lead to statistically significant improvements in glycemic control at 12 months. This suggests that in a well-resourced setting, standard care alone may be relatively effective. However, opportunities for further improvement remain. The lack of observed benefit may be due to process-related issues, such as suboptimal engagement with the intervention. Addressing these challenges should be a focus of future research.

## Supplementary material

10.2196/65919 Multimedia Appendix 1 Supplementary tables reporting patients’ baseline characteristics and their unadjusted and adjusted outcomes at 6 months.

## Abbreviations

HbA_1c_: glycosylated hemoglobin
MD: mean difference
MMC: Metabolic Management Center
OAD: oral antidiabetic drug
OR: odds ratio
SMBG: self-monitoring of blood glucose
T2DM: type 2 diabetes mellitus
