# Supplementary material for: Mobile App–Assisted Self-Monitoring of Blood Glucose in Type 2 Diabetes in Ningbo, China: 12-Month Retrospective Cohort Study
Source: JMIR Mhealth Uhealth. 2025 Sep 2;13:e65919. doi: 10.2196/65919 (PMC12404581; doi:10.2196/65919)
Supplement: Multimedia Appendix 1 [file mhealth-v13-e65919-s001.docx]

Supplementary Table 1: Baseline characteristics of patients before matching.

|  | **Mobile app-assisted SMBG group**  **n=80** | **Control group**  **n=1490** | **Absolute standardized difference (100%)** | ***P* value** |
| --- | --- | --- | --- | --- |
| **Sociodemographic characteristics** | | | | |
| Age, years (mean, SD) | 46.79 (10.04) | 50.64 (11.92) | 38.43 | .001 |
| Sex (n, %) | | | | |
| *Male* | 47 (58.75) | 953 (63.96) | 10.58 | .35 |
| *Female* | 33 (41.25) | 537 (36.04) | 10.58 |  |
| Education (n, %) | | | |  |
| *<High school* | 34 (42.50) | 727 (48.79) | 12.73 | .27 |
| *≥High school* | 46 (57.50) | 763 (51.21) | 12.73 |  |
| Household income (n, %) | | | | |
| *≤100,000 RMB* | 29 (36.25) | 550 (36.91) | 1.38 | .49 |
| *>100,000-300,000 RMB* | 30 (37.50) | 566 (37.99) | 1.01 |  |
| *>300,000 RMB* | 18 (22.50) | 263 (17.65) | 11.61 |  |
| *Unknown* | 3 (3.75) | 111 (7.45) | 19.47 |  |
| **Clinical characteristics** | | | | |
| Duration of T2DM, months (median, IQR) | 48.00 (5.00, 120.00) | 38.50 (15.50, 69.75) | 21.53 | .20 |
| Fasting blood glucose, mmol/L (mean, SD) | 9.20 (2.94) | 8.68 (2.94) | 17.70 | .12 |
| HbA1c, % (mean, SD) | 8.57 (2.33) | 8.06 (2.01) | 21.90 | .06 |
| Insulin regimen (n, %) | | | | |
| *No* | 59 (73.75) | 1072 (71.95) | 4.10 | .73 |
| *Yes* | 21 (26.25) | 418 (28.05) | 4.10 |  |

Abbreviations: SMBG self-monitoring of blood glucose, SD standard deviation, RMB Chinese Yuan, T2DM type 2 diabetes mellitus, IQR interquartile range, HbA1c glycosylated hemoglobin.

Supplementary Table 2: Unadjusted and adjusted outcomes at 6 months (62 in the mobile app-assisted SMBG group; 69 in the control group).

|  | **Unadjusted MD/OR** | **95% CI** | ***P* value** |  |
| --- | --- | --- | --- | --- |
| Fasting blood glucose (mmol/L) | -0.47 | -1.13, 0.20 | .17 |  |
| Fasting blood glucose <7mmol/L |  |  |  |  |
| *Control* | Ref |  |  |  |
| *Mobile app-assisted SMBG* | 1.19 | 0.58, 2.46 | .63 |  |
| HbA1c (%) | -0.47 | -0.92, -0.01 | .04 |  |
| HbA1c <7% |  |  |  |  |
| *Control* | Ref |  |  |  |
| *Mobile app-assisted SMBG* | 2.07 | 0.90, 4.75 | .09 |  |
|  | **Adjusted MD/OR** | **95% CI** | ***P* value** |  |
| Fasting blood glucose (mmol/L) | -0.45 | -1.11, 0.22 | .19 |  |
| Fasting blood glucose <7mmol/L |  |  |  |  |
| *Control* | Ref |  |  |  |
| *Mobile app-assisted SMBG* | 1.14 | 0.53, 2.46 | .74 |  |
| HbA1c (%) | -0.43 | -0.86, 0.00 | .05 | |
| HbA1c <7% |  |  |  |  |
| *Control* | Ref |  |  |  |
| *Mobile app-assisted SMBG* | 2.18 | 0.89, 5.37 | .09 |  |

Adjusted for age, sex, education, household income, duration of T2DM, baseline fasting blood glucose/HbA1c, and insulin regimen.

Abbreviations: MD mean difference, OR odds ratio, CI confidence interval, SMBG self-monitoring of blood glucose, HbA1c glycosylated hemoglobin.
